# The *Parauncinula polyspora* Draft Genome Provides Insights into Patterns of Gene Erosion and Genome Expansion in Powdery Mildew Fungi

**DOI:** 10.1128/mBio.01692-19

**Published:** 2019-09-24

**Authors:** Lamprinos Frantzeskakis, Márk Z. Németh, Mirna Barsoum, Stefan Kusch, Levente Kiss, Susumu Takamatsu, Ralph Panstruga

**Affiliations:** a Institute for Biology I, Unit of Plant Molecular Cell Biology, RWTH Aachen University, Aachen, Germany; b Plant Protection Institute, Centre for Agricultural Research, Hungarian Academy of Sciences, Budapest, Hungary; c Centre for Crop Health, University of Southern Queensland, Toowoomba, Australia; d Faculty of Bioresources, Mie University, Tsu, Japan; Cornell University

**Keywords:** genome evolution, plant pathogen, fungal genomics, transposable elements, repeat-induced point mutation

## Abstract

Due to their comparatively small genome size and short generation time, fungi are exquisite model systems to study eukaryotic genome evolution. Powdery mildew fungi present an exceptional case because of their strict host dependency (termed obligate biotrophy) and the atypical size of their genomes (>100 Mb). This size expansion is largely due to the pervasiveness of transposable elements on 70% of the genome and is associated with the loss of multiple conserved ascomycete genes required for a free-living lifestyle. To date, little is known about the mechanisms that drove these changes, and information on ancestral powdery mildew genomes is lacking. We report genome analysis of the early-diverged and exclusively sexually reproducing powdery mildew fungus Parauncinula polyspora, which we performed on the basis of a natural leaf epiphytic metapopulation sample. In contrast to other sequenced species of this taxonomic group, the assembled *P. polyspora* draft genome is surprisingly small (<30 Mb), has a higher content of conserved ascomycete genes, and is sparsely equipped with transposons (<10%), despite the conserved absence of a common defense mechanism involved in constraining repetitive elements. We speculate that transposable element spread might have been limited by this pathogen’s unique reproduction strategy and host features and further hypothesize that the loss of conserved ascomycete genes may promote the evolutionary isolation and host niche specialization of powdery mildew fungi. Limitations associated with this evolutionary trajectory might have been in part counteracted by the evolution of plastic, transposon-rich genomes and/or the expansion of gene families encoding secreted virulence proteins.

## Abstract (precis)

Powdery mildew fungi are widespread and agronomically relevant phytopathogens causing major yield losses. Their genomes have disproportionately large numbers of mobile genetic elements, and they have experienced a significant loss of highly conserved fungal genes. In order to learn more about the evolutionary history of this fungal group, we explored the genome of an Asian oak tree pathogen, *Parauncinula polyspora*, a species that diverged early during evolution from the remaining powdery mildew fungi. We found that the *P. polyspora* draft genome is comparatively compact, has a low number of protein-coding genes, and, despite the absence of a dedicated genome defense system, lacks the massive proliferation of repetitive sequences. Based on these findings, we infer an evolutionary trajectory that shaped the genomes of powdery mildew fungi.

## INTRODUCTION

Due to their ubiquitous presence in diverse environments with different intensities of selection pressure, fungi provide a unique insight into the evolution of eukaryotic genomes (1). The genomes of phytopathogenic fungi in particular have been in the spotlight because of their peculiar genome architectures (2), which foster mechanisms that allow for the rapid adaptation to an ever-changing plethora of host resistance genes (3). This high genome flexibility is considered to be a valuable feature for immune evasion, virulence, and long-term survival (4).

Powdery mildews (PMs) (Ascomycota, Erysiphales) are a monophyletic group of phytopathogens (5) that exclusively colonize living host plants—a lifestyle termed obligate biotrophy (6). Species of this family can have a broad or narrow range of hosts (7, 8), some of which include important agricultural and horticultural crops (9). Typically, PMs propagate via short (several days long) asexual life cycles and the production of conidiospores, but can undergo sexual propagation by the formation of ascospores under particular circumstances (e.g., for overwintering [10]).

PMs hold a special spot in filamentous plant pathogen genomics owing to the large size of their genomes, the vast amount of transposable elements (TEs) therein, and the large-scale loss of conserved fungal genes and associated cellular pathways (11–14). Recently, different laboratories have successfully managed to tackle technical challenges associated with the advanced genomic analysis of these pathogens: e.g., bottlenecks in extracting high-molecular-weight DNA (15) or in assembling complex repetitive genomes to the chromosome (arm) level (12, 16). Based on these methodological improvements, new information was provided on the population structure (13, 17), genome architecture (12, 13, 16, 18, 19), and evolution (20) of some of the species and their specialized forms (*formae speciales* [ff. spp.]). These studies suggest that TEs in PM genomes might be associated with the rapid turnover of virulence genes (encoding effectors) in the form of copy number variation (12, 16, 21). Additionally, it has been reported that PMs have a unique genomic architecture in which TEs and genes are intertwined, while other typical attributes found in TE-inflated genomes are missing: e.g., AT-rich isochores or large-scale compartmentalization (12, 16). Since Blumeria graminis has appeared relatively recently in the evolutionary history of PMs (20), the above-mentioned genome characteristics are likely to represent a contemporary step in their evolution. In order to understand how the genomes of these pathogens evolved and what acted as a substrate for their unique genome architecture, it would be necessary to seek information in the genomes of early-diverged PM species.

Here we present the draft genome of the PM species Parauncinula polyspora, a pathogen of the East Asian oak tree Quercus serrata. It has been estimated that species of the genus *Parauncinula*, which form a phylogenetic sister group to all other PMs (22), diverged from other genera of the Erysiphales 80 to 90 million years ago, rendering it one of the earliest-diverged PM genera known to date (22, 23). The four known species of *Parauncinula* (*P. curvispora*, *P. polyspora*, *P. septata*, and *P. uncinata*) differ from other PMs in their unique morphology and host range and also by lacking an asexual morph (i.e., conidiospores and conidiophores) (24). Our analysis, which is based on a natural leaf epiphytic metapopulation sample, reveals that *P. polyspora* has a surprisingly compact genome with a substantially smaller number of TEs than the more recently evolved PMs, a feature that cannot be attributed to the presence of conserved ascomycete genome defense mechanisms such as repeat-induced point (RIP) mutations. In addition, we report that the *P. polyspora* genome harbors a considerable number of conserved ascomycete genes (CAGs) that were subsequently lost in other PM lineages. Taken together, the presented analysis of the *P. polyspora* genome gives unexpected insights into the evolutionary history of PM fungi and provides broad suggestions on how TE inflation can affect the genomes of fungal phytopathogens.

## RESULTS

### Assembly of the *P. polyspora* genome from metagenomic *Q. serrata* samples.

*Parauncinula polyspora* is believed to be an obligate biotroph that relies on living plant tissues for growth and reproduction (22), and *in vitro* cultures of the species on artificial media have never been reported. Therefore, we resorted to sampling infected *Q. serrata* leaves harboring largely *P. polyspora* hyphae and ascomata (fruiting bodies) and sequenced the respective epiphytic metagenome. In order to avoid overcontamination, which could arise from sampling the entirety of the plant tissue, samples were prepared using cellulose acetate peelings (25) of the leaf epiphytic microbiota, and the respective genomic DNA was subjected to short-read sequencing. As expected, the initial data set contained sequences of a number of eukaryotic and prokaryotic taxonomic groups (see Fig. S1A in the supplemental material). Subsequently we assembled the respective short reads and followed a pipeline for stringent filtering to exclude both contaminating bacterial and eukaryotic sequences, assuming these are significantly less abundant than authentic *P. polyspora* sequences (see Fig. S1B [methods]). After filtering for bacterial sequences, two major populations of scaffolds could be separated based on k-mer depth only. One of the two, with approximately 30× coverage for each of its 1,321 scaffolds, contained sequences with similarity to PM fungi (average identity of 60% [Fig. S1C]), while the other (mostly with <5× coverage) contained a mixture of additional fungal and plant sequences. Among the filtered contigs of the first population, we identified a scaffold of extremely deep coverage (2,384×) that is identical to the deposited nucleotide sequence of the internal transcribed spacer (ITS) region for the *P. polyspora* specimen voucher MUMH4928 (see Fig. S2). DNA of this specimen was also sampled from PM-infected *Q. serrata* in the past (24).

10.1128/mBio.01692-19.1 FIG S1 Bioinformatic pipeline and quality control of the assembled sequences. (A) Taxonomic hit distribution of the original Illumina short reads based on the MG-RAST analysis by phylogenetic class. Hits with lower than 0.5% distribution were grouped as “Other.” (B) Assembly pipeline for the *P. polyspora* genome. Boxes signify bioinformatic steps; the number of scaffolds after key steps is given in blue font. (C) TBLASTN matches of coding sequences contained in the prefiltered *P. polyspora* scaffolds to the genomes of two powdery mildew species (*B. graminis* f. sp. *hordei* and *E. necator*) and an oak species (*Q. suber*) closely related to the host of *P. polyspora*. Each dot represents a TBLASTN hit, the *x* axis indicates scaffold length (bp), and the *y* axis indicates read coverage as derived by the SPAdes result. The identity of the respective TBLAST hits (% amino acid identity) is color coded according to the legend shown on the right. (D) Histogram of read depth per 1-kb window along the high-confidence contigs. (The number of bins is selected according to the Freedman-Diaconis rule.) Download FIG S1, PDF file, 1.0 MB. Copyright © 2019 Frantzeskakis et al. 2019 Frantzeskakis et al. This content is distributed under the terms of the Creative Commons Attribution 4.0 International license.

10.1128/mBio.01692-19.2 FIG S2 Multiple-sequence alignment of an ITS sequence-harboring scaffold with known ITS sequences of the genus *Parauncinula*. The multiple-nucleotide-sequence alignment was performed with CLC Sequence Viewer (QIAGEN) and comprises the assembled scaffold “NODE_26095_length_1823_cov_2384.869159” and the reference ITS sequences for *P. polyspora* (GenBank accession no. LC222318.1 and LC222319.1) and the related species *P. uncinata* (GenBank accession no. LC222314.1). Nonidentical nucleotides are shaded in black. Download FIG S2, PDF file, 1.3 MB. Copyright © 2019 Frantzeskakis et al. 2019 Frantzeskakis et al. This content is distributed under the terms of the Creative Commons Attribution 4.0 International license.

We then annotated these scaffolds using a previously developed pipeline for the barley PM fungus (12) and split them into 495 high- and 826 low-confidence scaffolds based on the relative frequency of leotiomycete-related annotations along each sequence (Fig. S1B [methods]). In the low-confidence group, 107 scaffolds, encompassing 10.9 Mb of sequence, always contained at least one gene with homology to Leotiomycetes and one or more with homology to extraneous species in the same scaffold, probably due to chimeric misassemblies. The remaining 719 low-confidence scaffolds (20.9 Mb of total sequence) contained either genes without any leotiomycete homology (18.4% of the scaffolds [19.0 Mb of sequence]) or no hits to the nonredundant (nr) database (81.5% of scaffolds [1.9 Mb of sequence]).

The resulting 495 high-confidence scaffolds contained 6,046 genes in 28.01 Mb of sequence. The read depth over 1-kb windows of these contigs is normally distributed and is on average 231× (Fig. S1D). Out of the annotated genes in these contigs, ∼97% have a detectable homolog in the Leotiomycetes (see Table S1A in the supplemental material). In terms of genome completeness, assayed using BUSCO (26), this assembly covers 90.75% of the common fungal gene space. Notably, the ratio of single-copy to duplicated BUSCO genes resembles that of other PM genome assemblies (Table S1B), indicating that our combined filtering method based on k-mer depth and sequence similarity has likely efficiently removed contaminating fungal sequences as well. Altogether, the careful epiphytic sampling method used here provided an enriched sample of *P. polyspora* sequences, while the availability of closely related PM genomes allowed the sifting of the scaffolds based on the homology of their annotations to PM genes. For the downstream analysis, we therefore used only the 495 high-confidence scaffolds and the predicted annotations they contained, knowing, however, that the rejected low-confidence scaffolds might also contain *P. polyspora-*related sequences (see above). Yet, the total amount of these (rejected) sequences is expected to represent only a small fraction of the genome according to the aforementioned BUSCO results, which are typically ∼97% for PM genome assemblies (12, 18).

10.1128/mBio.01692-19.8 TABLE S1 Features of the *P. polyspora* draft genome. This file lists various features of the *P. polyspora* genome, including a summary of Orthofinder results (Table S1A), BUSCO v1.22 (lineage “fungi”) results for the annotation of the *P. polyspora* genome (Table S1B), RepeatMasker v4.0.6 results for the high-confidence scaffolds of the *P. polyspora* genome (Table S1C), duplications in the *P. polyspora* and *B. graminis* f. sp. *hordei* DH14 genomes encoding secreted/nonsecreted proteins (Table S1D), SNPs based on remapping of the short reads on the high-confidence scaffolds (Table S1E), CAZyme prediction based on dbCAN v6 models (Table S1F), differences in the occurrence of Pfam domains between *P. polyspora* and *B. graminis* f. sp. *hordei* (Table S1G), and the number of SPs with SSF53933 domains (Table S1H). Download Table S1, XLSX file, 2.6 MB. Copyright © 2019 Frantzeskakis et al. 2019 Frantzeskakis et al. This content is distributed under the terms of the Creative Commons Attribution 4.0 International license.

**FIG 1:**
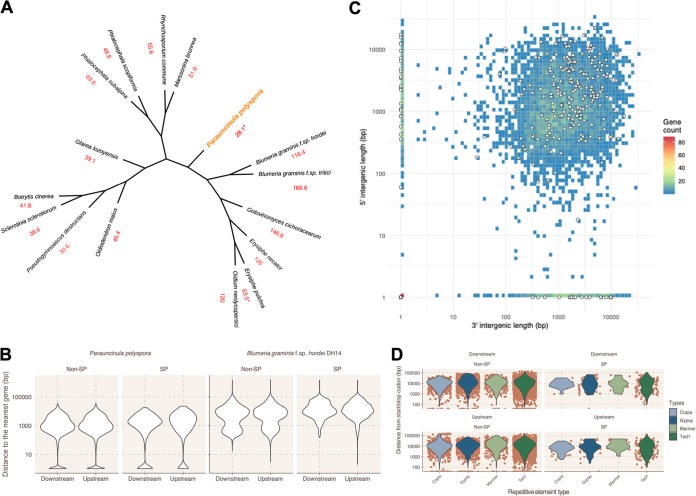
Characteristics of the *P. polyspora* draft genome. (A) The multilocus phylogeny (cladogram) of selected leotiomycete fungi, based on 1,964 single-copy orthologs identified by Orthofinder, was rendered by FastTree (ML Model: Jones-Taylor-Thornton) after alignment of the sequences with MAFFT. Bootstrap support is 100% for each node. Genome sizes are given below each species in Mb (in red). Draft PM short-read assemblies where an estimate of the total genome size is not known from the literature or otherwise are marked with an asterisk at the indicated genome size. (B) Violin plots illustrating the upstream (5′) and downstream (3′) intergenic length (*y* axis [bp]) of the *P. polyspora* SP- and non-SP-coding genes in comparison to the respective intergenic distances in *B. graminis* f. sp. *hordei*. (C) Gene density plot in relation to the 5′ (*y* axis) and 3′ (*x* axis) intergenic distances (bp) for *P. polyspora*. White circles depict SP-coding genes. The number of genes for a given intergenic length is color coded according to the legend on the right. (D) Violin plots illustrating the distance (*y* axis [bp]) of TEs from the transcriptional start/stop site of SP- and non-SP-coding genes. Orange dots indicate individual data points. The type of TEs is color coded according to the legend on the right.

To validate further the correct placement of this species as an early-diverged PM but also to corroborate the PM-related content of the high-confidence contigs, we proceeded with generating a multilocus phylogeny based on 1,964 single-copy orthologs of 16 sequenced Leotiomycetes (Fig. 1A). The placement of the species by this approach at the base of the PM clade is in accordance with previous results based on ribosomal DNA (rDNA) sequences (22).

### The compact *P. polyspora* genome lacks large-scale compartmentalization.

The assembled *P. polyspora* draft genome is very compact (∼28 Mb) compared to other sequenced PM species (Table 1), with a surprisingly small number of repetitive elements (∼8.5% [Table S1C]). In order to validate this observation, we used a k-mer-based approach (27) to calculate the genome size, which returned a similar estimate of 29.1 Mb. The difference in genome size from other sequenced PMs is also reflected by the length of the intergenic space, which is on average ∼3 to 4× smaller than that, e.g., in the case of *B. graminis* f. sp. *hordei* (Fig. 1B).

**TABLE 1:** *Parauncinula polyspora* draft genome assembly and annotation statistics compared to monocot (*B. graminis* f. sp. *hordei*)- and dicot (*E. necator*)-infecting PM species

Parameter	Result for:		
	*P. polyspora*	*B. graminis* f. sp. *hordei*^*a*^	*E. necator*^*b*^
Genome assembly			
Scaffolds, no.	495	99	5,935
Size, bp			
Min	966	16,042	498
1st quartile	17,297	52,946	1,297
Median	36,067	358,063	3,420
Mean	56,598	1,176,524	8,846
3rd quartile	74,488	1,602,884	11,116
Max	354,858	9,429,963	188,576
Total	28,016,241	116,475,897	52,505,057
*N* value, bp			
*N*_50_	98,897	3,906,310	21,433
*N*_90_	25,733	832,904	3,975
*N*_95_	17,861	443,704	2,006
Total repetitive content, %	8.5	74	63

Annotation			
Genes, no.	6,046	7,118	6,484
Avg size, bp	1,548	1,423	1,419
SPs, no.	261	805	607

a See reference 12.

b See reference 19.

We noted that secreted protein-coding genes (SP genes) do not constitute a separate compartment in the *P. polyspora* draft genome: i.e., there are no extended gene-rich/gene-sparse regions with an overrepresentation of SP genes (Fig. 1C; see Fig. S3 in the supplemental material). Moreover, the comparatively few transposable elements (TEs), which nevertheless comprise representatives of all major groups (retrotransposons, long terminal repeat [LTR] elements, and DNA transposons [Table S1C]), do not exhibit preferential insertion in the proximity of SP genes (<1 kb [Fig. 1D]). Finally, we also found that contrary to *B. graminis* f. sp. *hordei*, the *P. polyspora* draft genome has a lower ratio of duplicated genes coding for SPs and non-SPs (Table S1D).

10.1128/mBio.01692-19.3 FIG S3 Distribution and density of different genetic elements in the *P. polyspora* genome. The relative density (*y* axis) of genes encoding putative SPs, non-SP-coding genes, and TEs in the 10 largest scaffolds of the *P. polyspora* genome is plotted against the nucleotide position in the scaffold (*x* axis). Download FIG S3, PDF file, 0.2 MB. Copyright © 2019 Frantzeskakis et al. 2019 Frantzeskakis et al. This content is distributed under the terms of the Creative Commons Attribution 4.0 International license.

### The *P. polyspora* mating locus is indicative of homothallism.

Typically, PM fungi, like other Pezizomycotina, have one mating-type locus with two idiomorphs called *MAT1-1* and *MAT1-2* (28). The *MAT1-1* idiomorph encodes an α-domain protein and a high-mobility group (HMG) domain protein called MAT1-1-1 and MAT1-1-3, respectively. The *MAT1-2* idiomorph encodes only one HMG domain protein called MAT1-2-1 (12). Heterothallic PM fungi harbor either idiomorph in their genomes, while in rare instances of homothallism, *MAT1-2-1* and *MAT1-1-1* are present in the same genome (29). In our search of the *P. polyspora* draft genome, we identified only a single scaffold harboring mating-type genes (see Fig. S4A in the supplemental material), suggesting *P. polyspora* is a homothallic (self-fertile) PM species. These genes comprised a seemingly intact copy of *MAT1-2-1* and a likely pseudogenized copy of *MAT1-1-3* (Fig. S4B) residing ca. 10 kb away from each other on the same contig. We failed to identify a copy of *MAT1-1-1*, which is usually present in Leotiomycetes/PMs and in close physical proximity to *MAT1-1-3* (12, 30). Leotiomycete-homologous sequences representing this gene not only were lacking in the 495 high-confidence contigs but also were absent in the low-confidence contigs and the contigs with lower coverage assumed to represent contaminations. Additionally, the gene *SLA2*, which is typically found near the *MAT1* locus in many ascomycetes (30), resides on a separate scaffold, flanked by genes that are nonsyntenic to the canonical PM *MAT1* locus (Fig. S4A).

10.1128/mBio.01692-19.4 FIG S4 Analysis of the *P. polyspora* mating-type (*MAT*) locus. (A) Schematic representation of the *MAT* loci in *P. polyspora* and *B. graminis* f. sp. *hordei*. Genes functionally associated with the *MAT* locus are colored orange, pink, magenta, and blue, while flanking genes that are homologous and have a degree of synteny between the two species are shown in black. White arrows depict genes that are nonsyntenic but homologous. (B) Alignment of the putative *P. polyspora MAT1-1-3* pseudogene and its assumed functional *B. graminis* f. sp. *hordei* homolog (BGHR1_16067). Black arrowheads point to the positions of stop codons in the *P. polyspora* sequence. Sequence conservation (identical amino acid residues) is highlighted in red. Download FIG S4, PDF file, 0.4 MB. Copyright © 2019 Frantzeskakis et al. 2019 Frantzeskakis et al. This content is distributed under the terms of the Creative Commons Attribution 4.0 International license.

We also investigated the presence of single nucleotide polymorphisms (SNPs) and found that the majority of them are biallelic (98.2% [Table S1E]). Considering the likely homothallic nature of the species and taking into account that all PM genomes so far have been reported to be haploid during their vegetative growth phase, this finding indicates that our natural sample likely contained more than one *P. polyspora* isolate.

### The *P. polyspora* genome lacks evidence for the presence of RIP.

We proceeded in examining whether the compactness of the *P. polyspora* draft genome is due to the presence of the genome defense mechanism of RIP (31), which limits the spread of TEs and is absent in *B. graminis* f. sp. *hordei* and other sequenced PM species (6, 18, 19). We were unable to detect homologs of *Masc1*, *Masc2*, *Rid-1*, or *Dim-2* (GenBank accession no. AAC49849.1, AAC03766.1, XP_011392925.1, and XP_959891.1, respectively), which have been found to be associated with premeiotically induced DNA methylation in Ascobolus immersus and Neurospora crassa (32–35). Nevertheless, since these genes could have escaped the annotation process, or they could reside in genomic sequences that either had been removed during filtering or had not been fully assembled, we additionally searched for genomic sequences that bear characteristic RIP signatures (i.e., overrepresentation of certain dinucleotide repeats).

**FIG 2:**
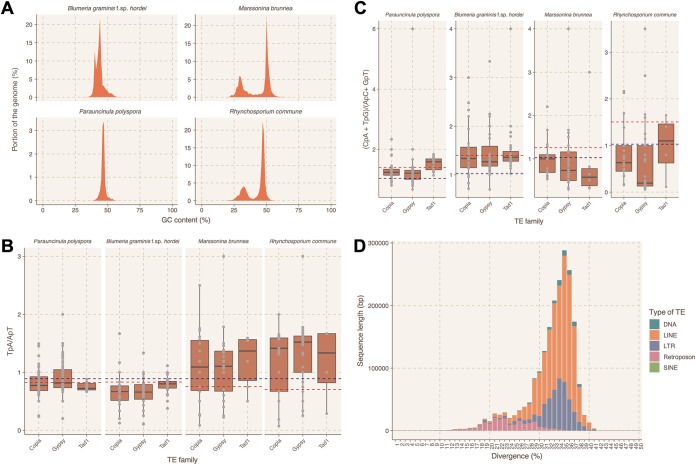
Analysis for signatures of RIP in the genomes of *P. polyspora* and related Leotiomycetes. (A) GC content profile of the *P. polyspora*, *B. graminis* f. sp. *hordei*, *M. brunnea*, and *R. commune* genomes. GC content (*x* axis [%]) is plotted against the respective portion of the genome (*y* axis [%]). (B and C) RIP index analysis for repetitive sequences of the four genomes. Shown as box plots are the TpA/ApT ratio (B, *y* axis) and the (CpA + TpG)/(ApC + GpT) ratio (C, *y* axis) for three different TE families (Copia, Gypsy, and Tad1 [*x* axis]). The blue line depicts the thresholds set from N. crassa (0.89 and 1.03, respectively [37]), while the red line indicates the threshold values obtained by the non-TE-containing genomic sequences of the respective genomes. (D) Divergence analysis for the TEs in the *P. polyspora* genome. The histogram illustrates the total sequence length (*y* axis [bp]) with a given nucleotide sequence divergence (*x* axis [%]) for different types of TEs according to the color code in the legend shown on the right.

Initially we explored whether the *P. polyspora* draft genome has AT isochores, a typical feature of RIP-containing genomes such as in the case of *Leptosphaeria maculans* (36). We found that neither the *P. polyspora* nor the *B. graminis* f. sp. *hordei* genome contains AT-rich isochores (Fig. S2A). However, we observed that the intensity of the AT signature in genomes of other Leotiomycetes that contain the genes necessary for RIP varies (see Fig. S5 in the supplemental material), with two exemplary cases for the presence of AT isochores being represented by Marssonina brunnea and Rhynchosporium commune (Fig. 2A). We proceeded by calculating two indices for these four genomes that could be informative regarding the presence of RIP. These two indices—TpA/ApT and (CpA + TpG)/(ApC + GpT)—have been used previously in N. crassa to detect signatures of RIP in repetitive sequences (37). They provide a measure of the prevalence and/or depletion of certain dinucleotides that are known results of RIP, while the respective baseline frequencies are calculated from nonrepetitive genomic sequences of the same genome. In the case of N. crassa, sequences with an TpA/ApT index higher than 0.89 and/or an (CpA + TpG)/(ApC + GpT) index lower than 1.03 suggest a biased frequency of AT dinucleotides caused by RIP (37). The baseline frequencies may vary between different species, as expected by the different overall nucleotide frequency of their genomes. In the genomes of *B. graminis* f. sp. *hordei* and *P. polyspora*, which seemingly lack RIP-related genes (see above), these indices likewise point to an absence of RIP. In contrast, respective values for *M. brunnea* and *R. commune* indicate the presence of RIP sequences, which is in agreement with the presence of AT-rich isochores and the genes of the RIP machinery in these genomes (Fig. 2B and C). Repetitive sequences that deviate from the set thresholds in *B. graminis* f. sp. *hordei* and *P. polyspora* could be relics of ancient RIP events, as has been previously suggested (38). Interestingly, the majority of the TEs in *P. polyspora* show high nucleotide sequence divergence (∼30 to 40% [Fig. 2D]), in contrast to *B. graminis* f. sp. *hordei*, where recent TE bursts have been observed, resulting in highly similar (>90% sequence identity) TE copies (12).

10.1128/mBio.01692-19.5 FIG S5 Presence/absence of AT isochores in the Leotiomycetes. Thirty-three leotiomycete genomes were assayed for the presence of AT isochores using OcculterCut v1.1. The portions of the genomes (*x* axis) with the respective GC content (*y* axis [%]) are plotted, indicating the presence of AT isochores in cases where a bimodal distribution is present (as for example prominently in Hymenoscyphus irciens). Download FIG S5, PDF file, 0.7 MB. Copyright © 2019 Frantzeskakis et al. 2019 Frantzeskakis et al. This content is distributed under the terms of the Creative Commons Attribution 4.0 International license.

### Powdery mildew genomes exhibit a lineage-specific loss of conserved ascomycete genes.

Next, we sought to determine if a set of conserved ascomycete genes (CAGs) that were previously found to be missing in the genome of the barley PM fungus (11) could be identified in the annotation of the *P. polyspora* draft genome. In addition, we surveyed additional PM, leotiomycete, and ascomycete proteomes to reevaluate the conservation of these proteins throughout the ascomycete lineage.

**FIG 3:**
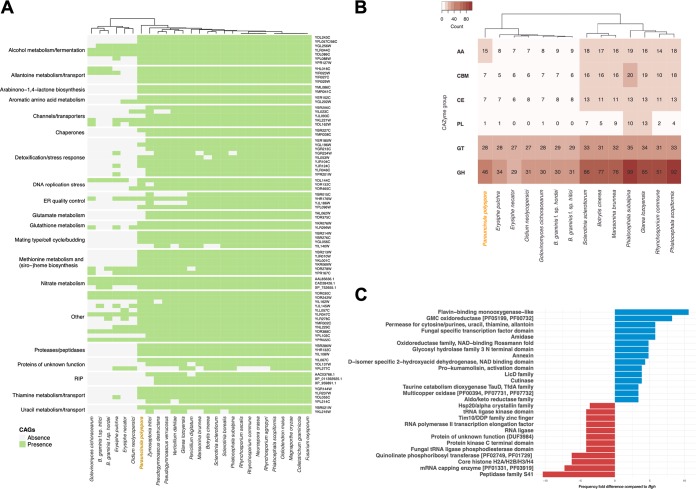
Presence and absence of conserved ascomycete genes (CAGs) in the genomes of PM. (A) Presence (green) and absence (gray) of CAGs in selected fungal species as cataloged previously (11). Hierarchical clustering of the respective species is indicated on the top, and Saccharomyces cerevisiae gene identifiers are given on the right. (B) CAZyme content of different leotiomycete species in comparison to *P. polyspora.* Abbreviations for CAZyme groups given on the left are as follows: AA, auxiliary activities; CBM, carbohydrate-binding modules; CE, carbohydrate esterases; PL, polysaccharide lyases; GT, glycosyl transferases; GH, glycoside hydrolases. CAZyme frequency per species is color coded according to the legend in the top left corner. In both panels A and B, the cladograms are based on hierarchical clustering. (C) Fold difference in frequency (*x* axis) in the occurrence of identified Pfam domains (*y* axis) in the predicted *P. polyspora* proteome in comparison to the predicted *B. graminis* f. sp. *hordei* proteome. Positive values are indicated by blue columns and negative values by red columns. Only differences of >3 between the two proteomes were considered for this graph.

Interestingly, a major portion of these genes (61 out of 82) could be detected in the *P. polyspora* draft genome (Fig. 3A), in contrast to other PMs in which the presence of these genes is low but somewhat variable. For example, in the *B. graminis* f. sp. *hordei* genome, only 16 out of 82 CAGs were found. The presence of 16 genes that were considered to be absent in the early versions of the *B. graminis* f. sp. *hordei* reference genome (11) might be explained by its recently improved assembly and annotation (12). Some of the genes found to be present in *P. polyspora* are critical for common biochemical pathways such as glutathione metabolism (Fig. 3A). However, the preservation of genes in *P. polyspora* does not apply to all otherwise widely conserved functional modules. For example, the RIP mechanism is lacking (see above), and the number of genes encoding carbohydrate-active enzymes (CAZymes), which are abundant in close relatives of PMs (e.g., in Botrytis cinerea and Sclerotinia sclerotiorum), is comparatively low (Fig. 3B; Table S1F). Notably, the results of this analysis indicate that the loss of some of these conserved genes might have happened independently for the different PM sublineages (e.g., for the homologs of YHL016C and YIR023W or for YGL202W [Fig. 3A]), while others were seemingly lost prior to the diversification of the PMs (e.g., for the homologs involved in glutamate metabolism [Fig. 3A]). In order to reduce the likelihood of false positives originating from the integration of sequences from other fungal species in the assembly, we also inspected the origin of the putative *P. polyspora* CAGs by BLAST analysis with the nr database. All returned with a best hit to leotiomycete sequences.

In addition, we found that beyond a number of unique Pfam-annotated functional domains that can be detected in the genome of *P. polyspora* compared to that of *B. graminis* f. sp. *hordei* (Table S1G), there is a considerable fold difference (>3) in the presence of 27 Pfam domains in *P. polyspora* compared to *B. graminis* f. sp. *hordei* (Fig. 3C). Particularly noteworthy in this context are the flavin-binding monooxygenase-like and glucose-methanol-choline (GMC) oxidoreductase functional domains, which each show an >8-fold-increased presence in relation to *B. graminis* f. sp. *hordei* (Fig. 3C). In contrast, in comparison to the *B. graminis* f. sp. *hordei* genome the *P. polyspora* genome appears to be depleted for genes encoding members of the peptidase family S41 (>10-fold-lower content than in *B. graminis* f. sp. *hordei* [Fig. 3C]). This comparison also emphasizes some of the unique aspects of the *B. graminis* f. sp. *hordei* genome, such as the expansion of genes encoding proteins with Sgk2 domains (superfamily SSF56112 [39]), which cannot be observed to the same extent in the other PM genomes (see Fig. S6 in the supplemental material).

10.1128/mBio.01692-19.6 FIG S6 Expansion of Sgk2-type serine-threonine/tyrosine-protein kinases in *Blumeria*. Heat map of orthogroups containing proteins with the Sgk2 domain (superfamily SSF56112). Numbers indicate the abundance of proteins of the respective orthogroup in the respective fungal species. Protein quantity is color coded according to the legend shown. Download FIG S6, PDF file, 0.3 MB. Copyright © 2019 Frantzeskakis et al. 2019 Frantzeskakis et al. This content is distributed under the terms of the Creative Commons Attribution 4.0 International license.

### *P. polyspora* has a compact predicted secretome with a low number of RNase-like SPs.

**FIG 4:**
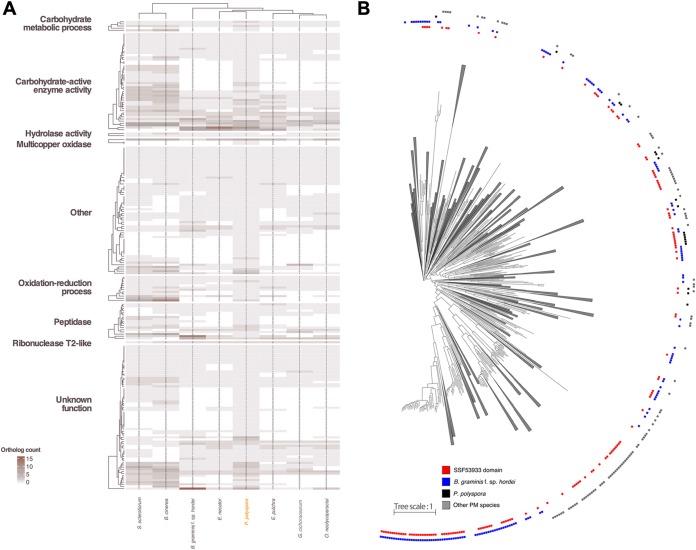
Comparative analysis of the *P. polyspora* secretome. (A) Heat map depicting the differences in the SP gene content of the publicly accessible PM genomes and the genomes of two related leotiomycete species, *S. sclerotiorum* and *B. cinerea*, in comparison to *P. polyspora* according to the color code shown in the bottom left corner. The description of the functional categories of the orthogroups is based on Pfam. The cladograms are the result of hierarchical clustering. (B) Maximum likelihood phylogenetic tree (phylogram) of 1,227 putative SPs with no Pfam annotation from the predicted proteomes of *B. graminis* f. sp. *hordei*, *P. polyspora*, *E. necator*, Erysiphe pulchra, Oidium neolycopersici, and Golovinomyces cichoracearum. Branches that do not contain any RNase-like domain-containing proteins (superfamily SSF53933) were collapsed. The category “other PM species” indicated by gray boxes includes *E. necator*, *E. pulchra*, *O. neolycopersici*, and *G. cichoracearum.* Branches with bootstrap values lower than 60% were trimmed.

We identified 261 SP candidates, out of which 193 had and 68 lacked a Pfam functional annotation. This is a surprisingly low number compared to *B. graminis* f. sp. *hordei* (805 SP candidates, 166 with and 639 without Pfam functional annotation), although other PM species that infect dicotyledonous plant species also have more compact secretomes (450 to 500 predicted SPs [18, 40]). However, we cannot exclude the possibility that this comparatively low number reflects, in part, the limitations of our annotation pipeline to predict gene models for SPs, which often lack sequence relatedness to known proteins, in the absence of transcriptomic data. Out of these 261 predicted SPs, only 70 lacked homology to annotated proteins in other PM genomes, and merely three (two lacking a Pfam functional annotation) appear to be unique to *P. polyspora*. Regardless of whether a known domain could be identified, several *P. polyspora* SPs have a homolog in *S. sclerotiorum* and *B. cinerea*, but not in other PMs (Fig. 4A), suggesting that these secretome members are dispensable and/or were lost in the course of the adaptation of PM fungi to new hosts. Notably, a sialidase domain-harboring SP (PARAU_11535; homologous to BLGH_03611) seems to be exclusively shared between PMs and appears to be absent in other Leotiomycetes, suggesting this could be an overall conserved virulence-related protein in PMs.

Genes encoding RNase-like candidate effector proteins are present in high numbers in the *B. graminis* f. sp. *hordei* genome (12, 41). We thus investigated if the predicted secretome of *P. polyspora* likewise contains RNase-like domain-carrying proteins (InterPro accession no. SSF53933, PF06479, and PF00445) and whether there is a potential phylogenetic relationship of such proteins with respective homologs in other PMs. We identified only two proteins carrying a recognizable RNase-related domain, which is a surprisingly low number compared to *B. graminis* f. sp. *hordei* (86 members [12]). We additionally searched for gene models that were excluded from the final annotation (*ab initio* unsupported calls), and we were able to identify 13 additional genes coding for RNase-like SPs (domain accession no. SSF53933), which again is still a substantially lower number than in *B. graminis* f. sp. *hordei*. In the other non-*Blumeria* PM species, a similarly low number of secreted RNase-like proteins can be identified (3 to 19 members [Table S1H]), which suggests that there might be a *Blumeria*-specific expansion of this gene/protein family. Interestingly, after ortholog clustering of the mature (signal peptide removed) peptide sequences of the SPs, additional candidate SPs were found to exhibit sequence similarity to these proteins, despite not having a recognizable RNase-like domain (see Fig. S7 in the supplemental material).

10.1128/mBio.01692-19.7 FIG S7 Orthogroup clustering of RNase-like SPs. Predicted powdery mildew SPs were clustered based on their mature (signal peptide removed) peptide sequence using Orthofinder. The heat map depicts the abundance of proteins for every orthogroup containing at least one SP with an RNase/RNase-like domain (SSF53933). Protein quantity is color coded according to the legend shown on the right. Download FIG S7, PDF file, 0.3 MB. Copyright © 2019 Frantzeskakis et al. 2019 Frantzeskakis et al. This content is distributed under the terms of the Creative Commons Attribution 4.0 International license.

In nearly all PM species examined here, family-specific expansions of genes encoding RNase-like SPs can be observed, and in addition, these RNase-like proteins are spread throughout different orthogroups (Fig. S7), suggesting a polyphyletic origin. We generated a maximum likelihood phylogenetic tree using all PM SP candidates with no Pfam domain (Fig. 4B), which further supports the notion that these RNase-like SPs are very diverse and potentially not of monophyletic origin. However, it has been recently suggested that despite the fact that they exhibit a severely eroded primary amino acid sequence similarity, the respective proteins may share an ancestor, as evidenced by a conserved intron position in the respective genes (42).

## DISCUSSION

### A natural leaf epiphytic metapopulation sample permits the assembly of a complex eukaryotic draft genome.

As a member of the taxonomic group of PM fungi, *P. polyspora* is believed to be an obligate biotrophic organism that cannot be cultured *in vitro*. Since the fungus is a pathogen of a tree species (*Quercus serrata*) that is native to eastern Asia, its propagation in pure culture would represent a formidable task. We thus took advantage of natural *P. polyspora*-infected leaf samples in the context of our project. Cellulose acetate peelings captured the leaf epiphytic microbiota of these samples and enabled the enrichment of sufficient biomass to allow genomic DNA extraction, sequencing, and assembly of a *P. polyspora* draft genome comprised of 495 high-confidence scaffolds (Fig. S1B). Although this assembly is based on a natural microbial metapopulation, k-mer and read depth coverage (Fig. S1C and D), sequence relatedness of the annotated genes (Table S1A), results of the BUSCO analysis (Table S1B), and the outcome of a multilocus phylogeny (Fig. 1A) support the notion that our assembly is of sufficient completeness and quality to allow downstream analyses. Inherent to studies based on natural metapopulation samples, we acknowledge that we cannot fully rule out contaminations and assembly artifacts, which to some extent limits the explanatory power of our study.

### Coevolutionary pace and life cycle attributes might drive genome plasticity in PMs.

Little is currently known about the biology of *P. polyspora*, which seems to propagate exclusively via ascospores produced during sexual reproduction, lacking a recognized asexual morph (i.e., conidiophores and conidia [22, 24]) and thus the asexual part of the typical PM life cycle. Accordingly, its precise host range, its mode of infection, the duration of its life cycle, and whether it represents indeed a homo- or heterothallic species remain to be explored.

At ∼29 Mb, the assembled draft genome of this species is approximately 4-fold smaller than the average genome of other sequenced PM fungi (12, 16, 18, 19) and even smaller than the average filamentous ascomycete genome (∼37 Mb [43]). Accordingly, it lacks the distinct abundance of repetitive elements that otherwise characterizes genomes of PM species (Table S1C), but shares with them a comparatively low gene number (∼6,000) and a noncompartmentalized organization (Fig. 1C; Fig. S3) (12). These inferences derive from the analysis of the assembled high-confidence contigs obtained from our metagenomic *Q. serrata* sample and thus, in principle, could reflect a considerable underrepresentation of the *P. polyspora* genome. The number of repetitive elements could also be underestimated, as this happens even in assemblies of “streamlined” genomes (44). However, results of k-mer analysis support the estimated genome size via an independent approach, and the low average size of the intergenic space (Fig. 1A) additionally corroborates the idea of a very compact genome. Furthermore, gene number and results of the BUSCO analysis indicate that the majority (∼94%) of the typical PM gene space is covered by the high-confidence contigs. Even when taking into account the low-confidence contigs, the calculated genome size would not exceed 61 Mb—a value still 2-fold smaller than that of other sequenced PM fungi.

Notably, the draft genome is indicative of homothallism (self-fertilization) since we located characteristic genes of both mating types (MAT1-1 and MAT1-2) on a single contig (Fig. S4). This finding is surprising since the majority of PMs are considered to be heterothallic (29), and therefore we expected that our sampling material containing sexual reproduction structures (chasmothecia) should recover discrete scaffolds representing both mating-type loci. Nevertheless, homothallism in PM fungi has been reported several times (29), although most of these reports lack molecular evidence. In contrast to the described homothallic Plantago lanceolata pathogen Podosphaera plantaginis, where both *MAT* idiomorphs likely exist as functional genes within the same genome (29), in *P. polyspora* the recovered *MAT* locus contains a seemingly intact *MAT1-2-1* and an apparently pseudogenized *MAT1-1-3* (Fig. S4). Moreover, a homolog of *MAT1-1-1*, supposed to be an indispensable feature of the MAT1-1 mating type (30), seems to be completely absent. Thus, in *P. polyspora*, a joint *MAT1-1*/*MAT1-2* locus exists, but it appears as if one of the two idiomorphs (*MAT1-1*) became dispensable for sexual reproduction of this fungus. This scenario, where only one MAT idiomorph is sufficient for self-fertility, has been observed previously in ascomycetes (“same-sex mating” [45]), yet it is considered a rare occurrence (46). As in other homothallic ascomycetes and their closely related heterothallic counterparts (47), synteny between the *P. polyspora MAT1-1*/*MAT1-2* locus and the *MAT* loci of the closely related heterothallic *B. graminis* f. sp. *hordei* is poorly conserved. This reported lack of synteny in homothallic loci compared to closely related heterothallic species might also explain why the arrangement of the *P. polyspora* locus is different from the suggested locus of *P. plantaginis* (29).

Interestingly, the *P. polyspora* genome retained many genes that were lost in more recently evolved PMs, but similarly to the latter lacks genes associated with the RIP genome defense mechanism (Fig. 2A to C and Fig. 3A). This finding suggests that the RIP pathway was abandoned in an early progenitor species of the PM lineage at least 80 to 90 million years ago prior to the separation of the *Parauncinula* genus from the other PMs (23). The absence of RIP but maintenance of a compact genome in the case of *P. polyspora* implies that the propagation of TEs in the genomes of the Erysiphales might be restrained by other mechanisms and/or suppressed by certain attributes of the *Parauncinula* life cycle. A plausible hypothesis is that the sexual propagation in *Parauncinula* might contribute to the maintenance of a lean genome, in comparison to the mainly asexually propagating species of the genera *Golovinomyces*, *Blumeria*, *Podosphaera*, and *Erysiphe* (10). This idea would support the overall assumption that sexual recombination can limit the uncontrolled proliferation of TEs while at the same time help in spreading beneficial mutations (48). Nevertheless, examples of fungal species lacking sexual propagation, RIP, and an abundance of TEs exist (49), indicating that broad generalizations cannot be easily made.

On the other hand, it might be argued that the selection pressure in the *P. polyspora-Q. serrata* interaction is comparatively low. In cases where PMs infect annual, agriculturally relevant hosts, breeding and growing of cultivars with novel resistance specificities as well as crop protection measures place massive selection pressure on pathogen populations. A particularly plastic and rapidly evolving genome (opposed to the case of *P. polyspora*) should be an advantage during adaptation and survival of crop pathogens. *P. polyspora* likely causes monocyclic infections with a much lower propagation rate, since the lack of conidiophores does not allow for the rapid and profuse aerial dispersal of conidia and multiple infection cycles per year—as, for example, in *B. graminis*. In addition, the host, *Q. serrata*, has a long, perennial life cycle, leading to a limited ability to evade a pathogen with a rapid turnover of resistance genes. Also, its genetically diverse local populations (50) may offer a less selective environment than the hosts in uniform agricultural settings (51, 52). In this scenario, both partners (*P. polyspora* and *Q. serrata*) might be locked in an arms race, albeit at a much slower pace compared to the interactions between many annual plant species and the respective PMs. This could be echoed by the *P. polyspora* genome, in which the relative scarcity of TEs does not offer a template for rapid evolution of virulence genes by duplications (Table S1D), small-scale rearrangements, or deletions, as has been proposed for *B. graminis* f. sp. *hordei* and *B. graminis* f. sp. *tritici* (12, 16, 53). The smaller secretome and the number of unique secreted proteins with no known functional domains (i.e., typical effector candidates) in *P. polyspora* can also be considered as support for this hypothesis. While definitely this result originates in part from the lack of transcriptomic data, it is now a more general observation that dicot-infecting PM have more compact secretomes, presumably because of different selection processes than those acting on the grass-infecting species (i.e., negative versus positive selection [40]). Our data, together with results from recent studies on genomes of additional dicot-infecting PM species (18, 19, 40), indicate that the expansion of the total amount of virulence genes or particular families thereof might be specific to PM lineages that emerged recently and are under high selection pressure.

### Gene functional reduction and family expansion are an ongoing process in PMs.

The *P. polyspora* draft genome reveals that more CAGs are present in an ancestral than in derived PM species and also that the respective gene losses are unequal (Fig. 3A). Recent studies indicate that this likewise extends to CAGs not included here (18), as for example, shown by the absence of a part of the RNA interference (RNAi) machinery in the grapevine PM pathogen Erysiphe necator (54). Since the remaining CAGs are dispersed in the genome, it is unlikely that their absence is the result of some large-scale genome reduction: e.g., the loss of a single chromosome. Instead, it seems to be in part stochastic, possibly due to local illegitimate recombination activity caused by TE insertions adjacent to the genes (53). The eventual fixation of these losses as observed in the different PM lineages could be a driver of the subsequent strict association of these species with a low number of specific hosts and their spatial and reproductive isolation from other PMs. It may also favor the succeeding loss of additional functional categories (Fig. 3B and C and Fig. 4A) that are related to virulence (i.e., peptidases, CAZymes, and redox enzymes) but no longer needed to that extent in a highly specialized host environment. This might also apply to asexual reproduction (conidiospore formation), which appears to be absent in species of the genera *Parauncinula*, *Brasiliomyces*, and *Typhulochaeta* within the Erysiphales (10). Taken together, the pattern of present/absent genes in the PM genomes thus appears to result from a mixture of divergent, convergent, and individual gene losses.

We hypothesize that in PMs, extinction by genome erosion is avoided by the compensatory lineage-specific expansion of gene families that support virulence (e.g., encoding effector proteins) or other physiological processes. Two examples that were identified here are secreted RNase-like proteins (Fig. 4B; Fig. S7) and Sgk2-type serine/threonine protein kinases (Fig. S6), which appear to have specifically multiplied their numbers in *B. graminis* f. sp. *hordei*. Some RNase-like SPs have been previously shown to be involved in virulence and/or to be recognized by the plant host (17, 42, 53, 55–57), while the role of Sgk2-type serine/threonine protein kinases in PM biology remains elusive (39). Yet in other fungal pathogens, it is speculated that kineome expansion is related to environmental and stress responses (58).

### A hypothetical model for the evolutionary adaptation of powdery mildew fungi.

**FIG 5:**
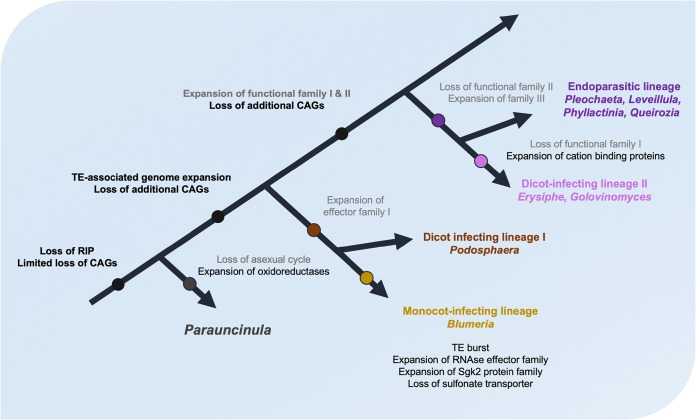
A hypothetical model for the evolution of the PM fungi. A simplified and schematic phylogenetic tree illustrating the evolution of PM fungi is shown. Genomic features for which some evidence is provided by the present work and previous studies are shown in black, while hypothetical losses and expansions are depicted in gray, and major/driving events for adaptation to new hosts are highlighted in boldface.

Based on the data available so far, we hypothesize the following scenario, which is summarized in Fig. 5. An ancestor of the PM lineage experienced the loss of the RIP machinery and a limited loss of CAGs. After diverging from the other PM lineages, *P. polyspora* was subject to a loss of the asexual life cycle, the establishment of homothallism, and the expansion of particular protein families (e.g., monooxygenases and oxidoreductases). The other PM genomes, derived after this early split, underwent a TE-associated genome expansion and the loss of additional CAGs. Separate lineages strictly associated with certain monocot or dicot hosts were established, and different modes of infection (epiphytic versus endophytic) evolved based on a reoccurring pattern of loss and/or expansion of virulence-related and other gene families. This pattern is exemplified by the specific expansion of RNase-like effector proteins and Sgk2 kinases in the monocot-infecting lineage, but also by the unequal loss of CAGs and the reported expansion of other functional families in dicot-infecting PMs (e.g., cation binding proteins [18]).

## MATERIALS AND METHODS

### Genomes and proteomes used in this study.

The genomes and proteomes used in this study are listed in Table 2.

**TABLE 2:** Genomes and proteomes used in this study

Data set	Accession no./release	Reference/website
Nonredundant protein database (nr)	Downloaded 28 August 2017	ftp.ncbi.nlm.nih.gov
UniProt	2016_05	http://www.uniprot.org/downloads
Aspergillus niger CBS 513.88	GCF_000002855.3	NCBI
Blumeria graminis f. sp. *tritici* 96224	GCA_000418435.1	NCBI
Blumeria graminis f. sp. *hordei* K1	GCA_000401615.1	NCBI
Blumeria graminis f. sp. *hordei* DH14	GCA_900239735.1	https://genome.jgi.doe.gov/Blugr2/Blugr2.info.html
Botrytis cinerea B05.10	GCF_000143535.1	NCBI
Colletotrichum graminicola M1.001	GCF_000149035.1	NCBI
Erysiphe necator	GCA_000798715.1	NCBI
Erysiphe pulchra	GCA_002918395.1	NCBI
Fusarium oxysporum	GCA_000149955.2	NCBI
Glarea lozoyensis 20868	GCF_000409485.1	NCBI
Golovinomyces cichoracearum	GSE85906 (GEO)	NCBI
Magnaporthe oryzae 70-15	GCF_000002495.2	NCBI
Marssonina brunnea f. sp. MBm1	GCF_000298775.1	NCBI
Neurospora crassa	p3_p13841	ftp://ftpmips.gsf.de/fungi/
Oidiodendron maius	GCA_000827325.1	NCBI
Oidium neolycopesici	GSE85906 (GEO)	NCBI
Penicillium digitatum	GCA_000315645.2	NCBI
Phialocephala scopiformis	GCF_001500285.1	NCBI
Phialocephala subalpina	GCA_900073065.1	NCBI
Pseudogymnoascus destructans	GCF_000184105.1	NCBI
Pseudogymnoascus verrucosus	GCF_001662655.1	NCBI
Quercus suber	GCF_002906115.1	NCBI
Rhynchosporium agropyri	GCA_900074905.1	NCBI
Rhynchosporium commune	GCA_900074885.1	NCBI
Rhynchosporium secalis	GCA_900074895.1	NCBI
Sclerotinia borealis	GCA_000503235.1	NCBI
Sclerotinia sclerotiorum 1980	GCF_000146945.1	NCBI
Verticillium dahliae VdLs.17	GCF_000150675.1	NCBI
Zymoseptoria tritici IPO323	GCF_000219625.1	NCBI

### Sampling and genomic DNA extraction.

Infected leaves of a single *P. polyspora-*infected *Q. serrata* tree were collected in 2017 in Torimiyama Park, Haibara, Uda-shi, Nara Prefecture, Japan (N34.543809, E135.944306). The leaf samples were dipped in 5% (wt/vol) cellulose acetate-acetone solution and then placed to dry. The cellulose was peeled off using forceps, and the sample was ground in liquid nitrogen with a mortar and pestle. The resulting cellulose fragments with fungal structures attached were transferred to 2-ml tubes, and genomic DNA extraction was performed as described in reference 15. Afterwards, small DNA fragments (<100 bp) were removed using AMPure XP beads (Beckman Coulter, Krefeld, Germany), and the quantity and quality of the DNA were assessed using a NanoDrop spectrophotometer (Thermo Fisher Scientific, Darmstadt, Germany) and a Qubit fluorometer (Thermo Fisher).

### Genome sequencing, assembly, and functional annotation.

Illumina library preparation (TruSeq DNA Nano; llumina) and genomic sequencing were performed by CeGaT GmbH in Tübingen, Germany. The library was sequenced on the NovaSeq 6000 platform and resulted in 163.1 million paired raw reads (2 × 150 bp, a total of 24.6 Gbp of data). The reads were assessed for their content of leotiomycete sequences using MG-RAST (MG-RAST identification no. f5bdd547896d676d343830363131372e33) (59).

The pipeline followed to assemble the genome is briefly presented in Fig. S1A. In more detail, the adapters were trimmed with Skewer (60) and then passed to BFC (-b 32 -k 25 -t 10) (61) for error correction and removal of singleton k-mers. The corrected reads were then assembled with SPAdes v3.11.1 (--only-assembler -k 31,51,71,91,111 --meta) (62). In order to remove bacterial and eukaryotic contaminating sequences from the resulting scaffolds, the sequences were initially searched by BLAST against a set of 3,837 plant-associated bacterial genomes (http://labs.bio.unc.edu/Dangl/Resources/gfobap_website/index.html; 63). The resulting scaffolds were filtered based on their depth (cutoff of >20× [Fig. S1C]) and the homology of their annotations to the Leotiomycetes. For the exclusion based on the leotiomycete homology, after the annotation (see below) the predicted genes were used for homology search against the NCBI nr protein database (last accessed November 2017) using BLAST+ v2.3.0 (64). Scaffolds where the two most frequent hits to the nr belonged to the Leotiomycetes were deemed as high-confidence scaffolds, while the rest were placed in the low-confidence group. The high-confidence scaffolds were assessed for coverage of the gene space using BUSCO v1.22 (26), and an additional size estimation based on k-mer abundance was provided using Jellyfish v2.2.10 (27) with reads that aligned only to the high-confidence contigs and a k-mer option for 31 bp (-m 31).

For the annotation of the scaffolds, we followed the same pipeline as described before (12) using MAKER (65). The data sets provided as evidence are listed in Table 2. Afterwards functional annotation was performed using InterProScan v5.19-58.0 (66) and HMMER v3.1 (67) with dbCAN v6 (68) for the identification of CAZymes specifically. Putatively secreted proteins with no transmembrane domains were identified using SignalP v4.1 (69) and TMHMM v2.0c (70). Mating-type genes were identified by bidirectional BLAST searches (64) using BLASTP and TBLASTN with an E value cutoff of 10e−5.

### Analysis of repetitive sequences.

Repetitive sequences were identified using RepeatMasker v4.0.6 (http://www.repeatmasker.org) using Repbase as a database (last accessed 9 June 2016). Subsequently, a repeat landscape was generated for *P. polyspora* as described before (12). GC composition of the selected leotiomycete genomes and dinucleotide frequencies were calculated using OcculterCut v1.1 (71) and RIPCAL v2 (72), respectively.

### Orthogroup inference, phylogeny, and nucleotide polymorphisms.

Identification of ortholog groups and generation of gene family trees were performed using OrthoFinder v1.1.2 (73). The maximum likelihood phylogenetic trees based on putatively secreted proteins with no Pfam annotation or on single-copy orthologs were generated using FastTree v2.1.10 (74) after alignment of the protein sequences with MAFFT v7.310 (75). Figures of the trees were generated using iTOL (76) and are available at https://itol.embl.de/shared/lambros2. CAG search using the proteomes listed above was performed using BLASTP (64) with an E value threshold of 10e−5.

In order to discover the number of single nucleotide polymorphisms in the *P. polyspora* genome assembly, we initially mapped the reads using BWA-MEM v0.7.15-r1140 (77). The resulting sam file was processed (conversion to bam, sorting) with Picard tools v2.8.2 (http://broadinstitute.github.io/picard), and then polymorphisms were identified using samtools mpileup and bcftools (v0.1.19) (78) and filtered with SnpSift v4.3i (QUAL >= 20 && DP > 3 && MQ > 50) (79).

### Data availability.

Corresponding R scripts and associated files (phylogenetic trees, tables, etc.) used for generating figures used in this article have been deposited in GitHub at https://github.com/lambros-f/paraun_2018. In all software utilized for the analysis, if no explicit settings are mentioned, then the defaults were used. The data set, including raw reads and the assembled genome used here, has been deposited in the European Nucleotide Archive (ENA) under accession no. PRJEB29715.
