# Supplementary figures and images for: The Parauncinula polyspora Draft Genome Provides Insights into Patterns of Gene Erosion and Genome Expansion in Powdery Mildew Fungi
Source: mBio. 2019 Sep 24;10(5):e01692-19. doi: 10.1128/mBio.01692-19 (PMC6759760; doi:10.1128/mBio.01692-19)

**A**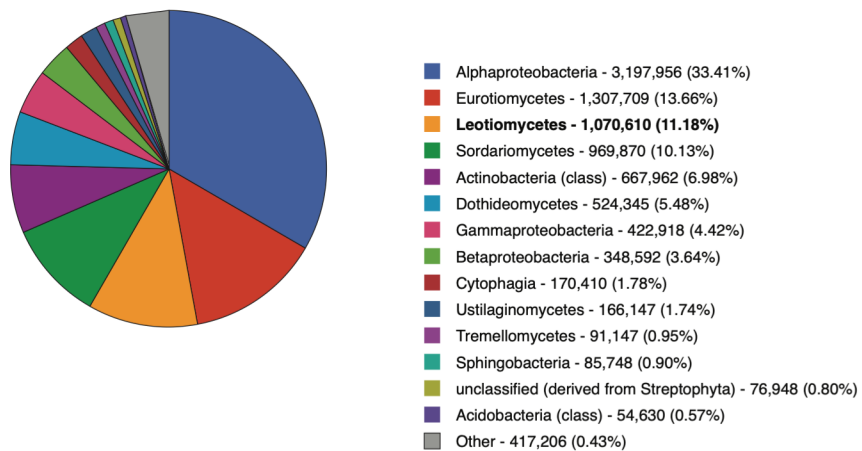**B**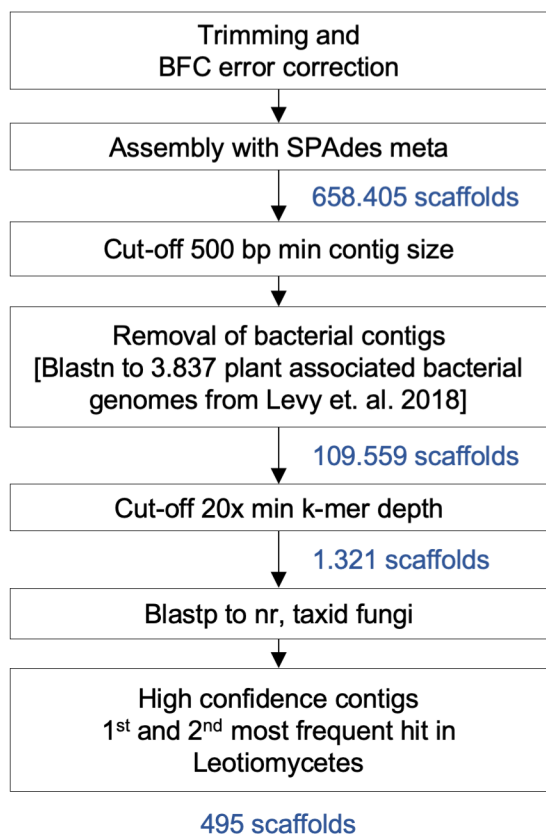**C**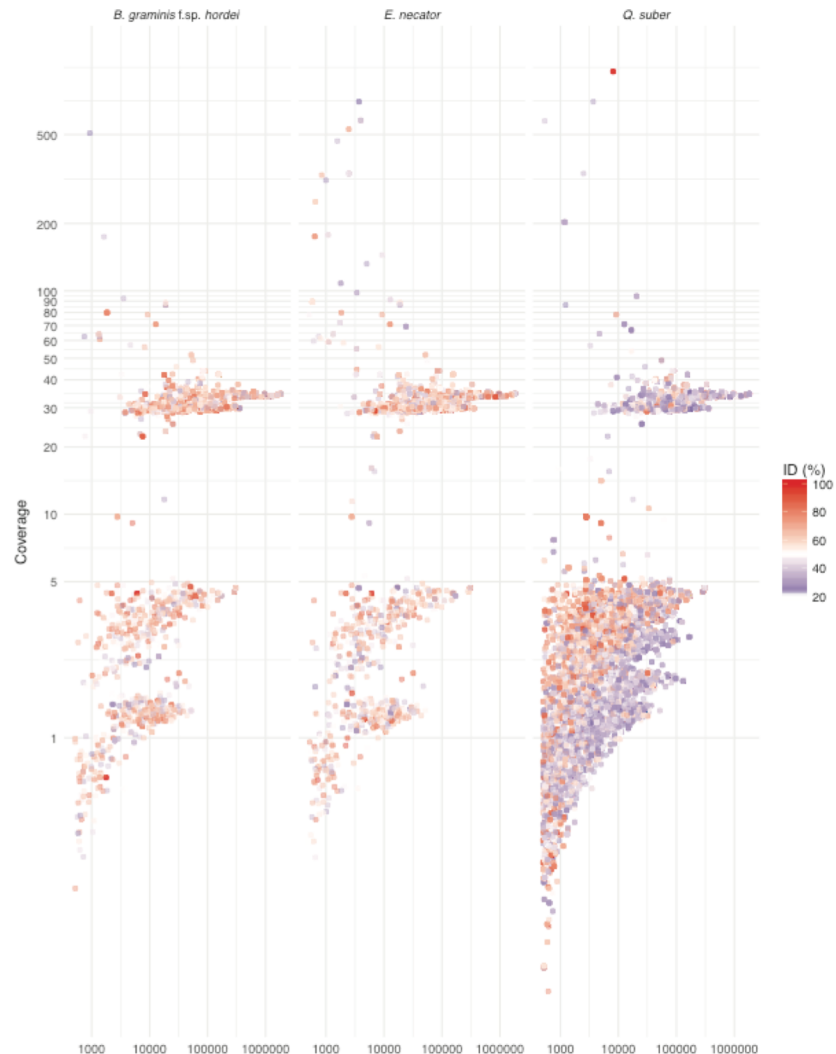**D**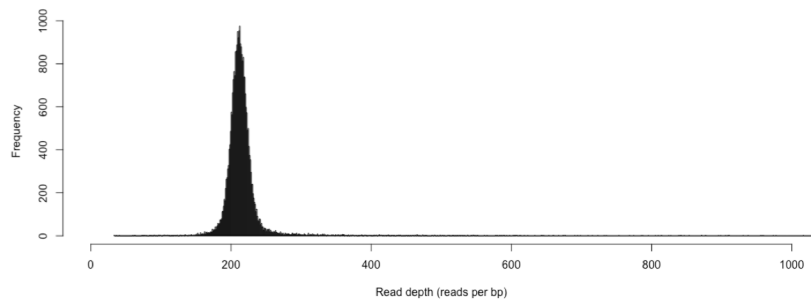

Supplement: FIG S1 [file mBio.01692-19-sf001.pdf]

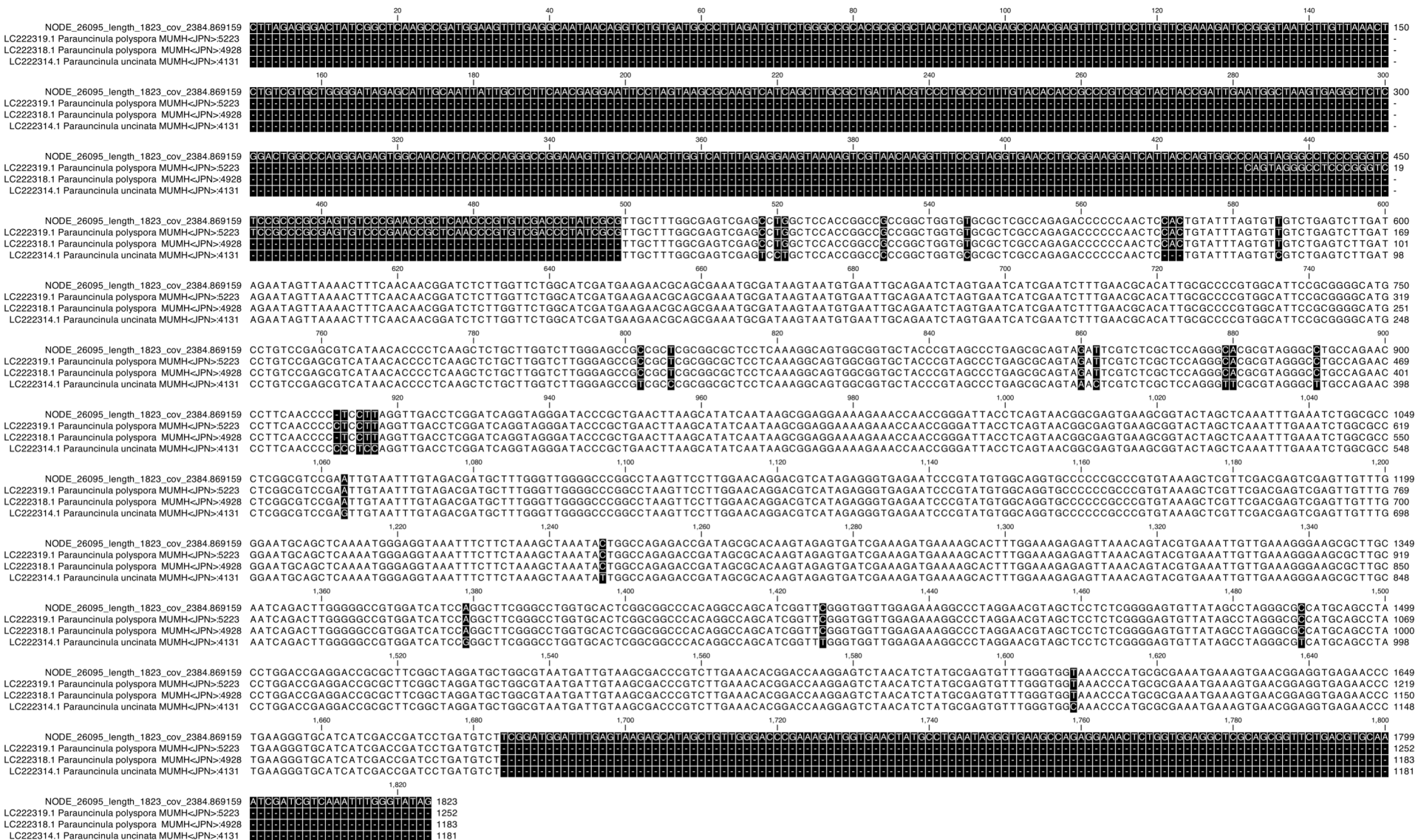

Supplement: FIG S2 [file mBio.01692-19-sf002.pdf]

A

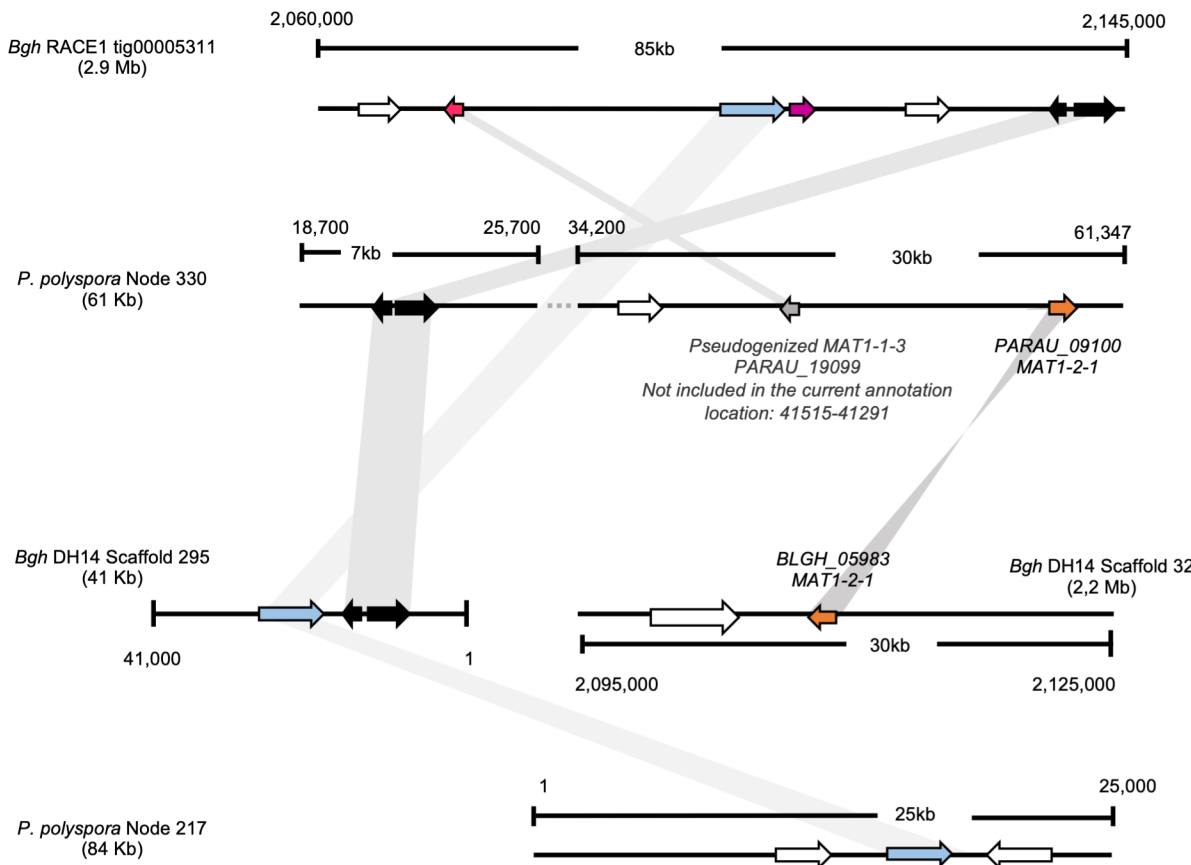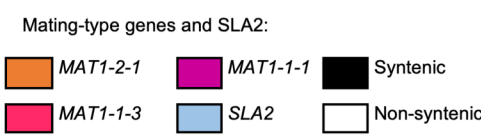

B

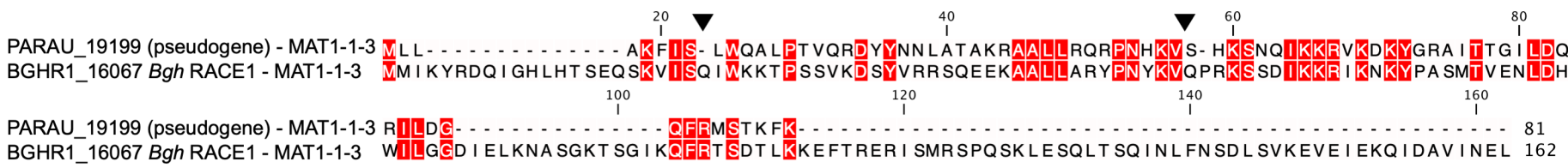

Supplement: FIG S4 [file mBio.01692-19-sf004.pdf]

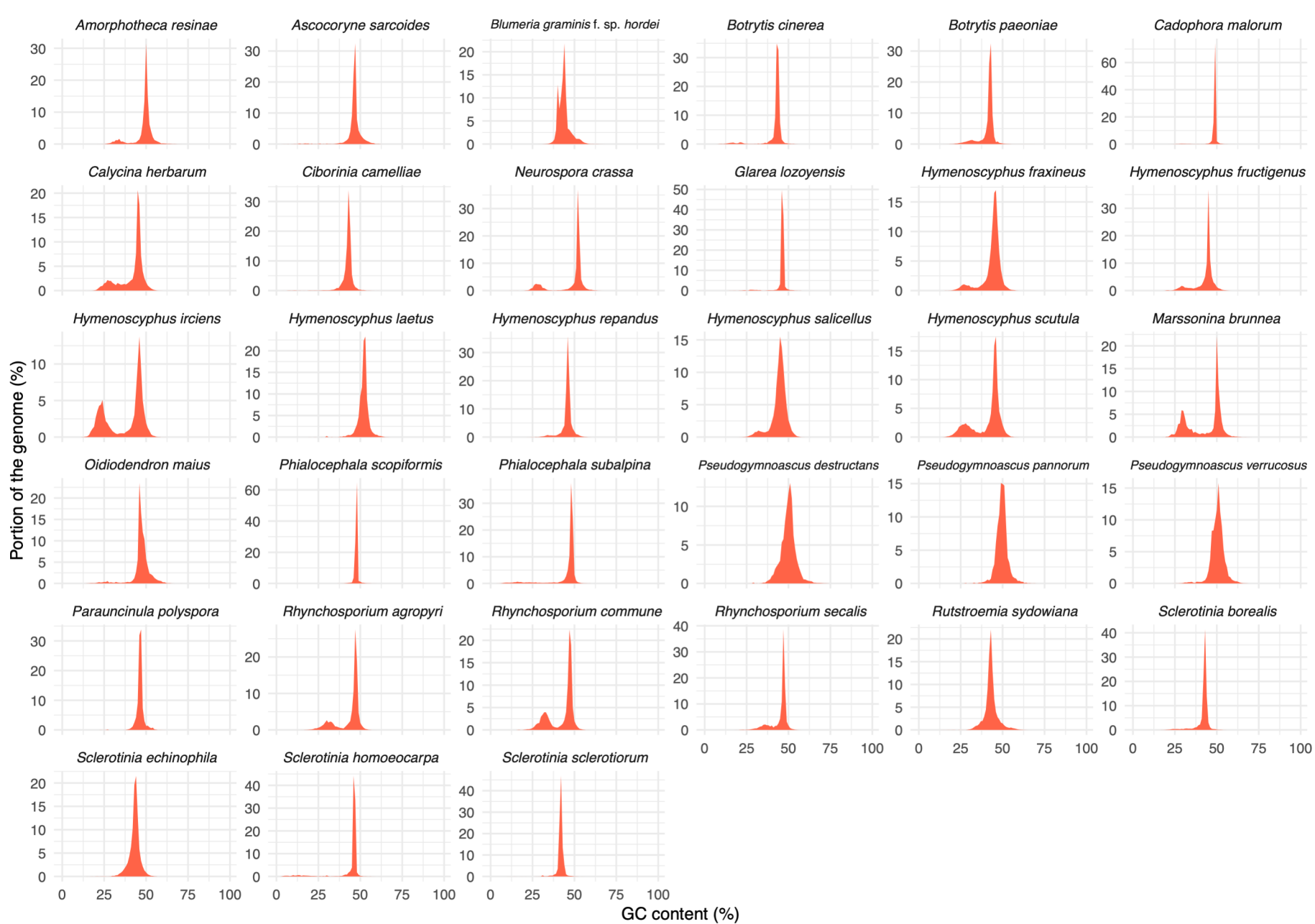

Supplement: FIG S5 [file mBio.01692-19-sf005.pdf]

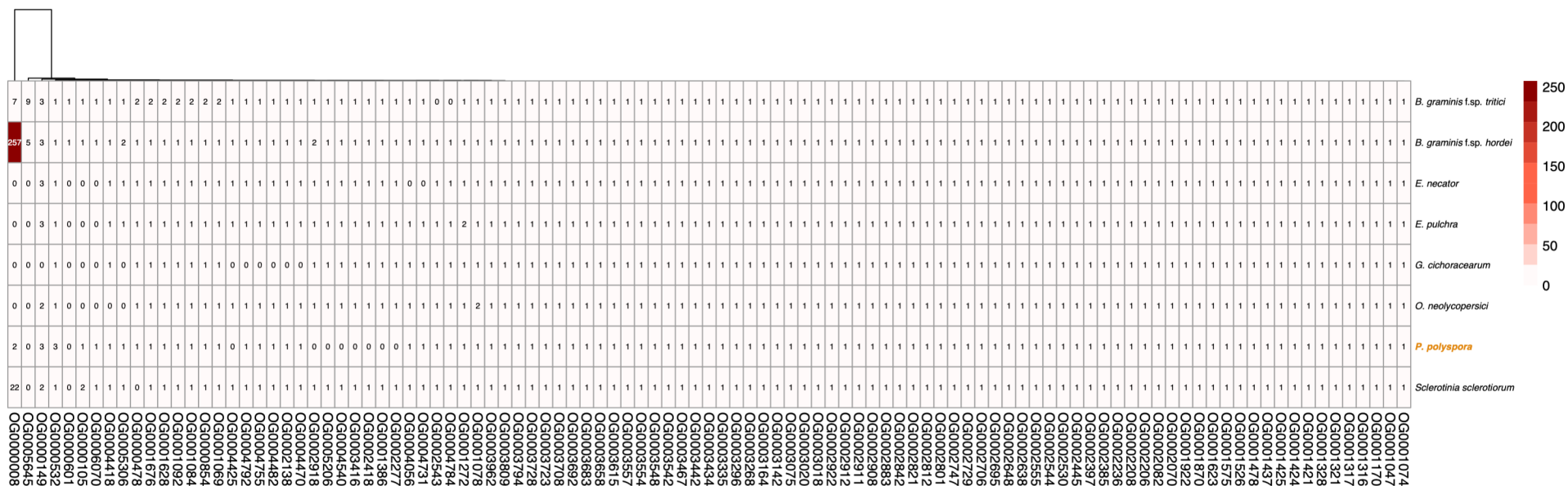

Supplement: FIG S6 [file mBio.01692-19-sf006.pdf]

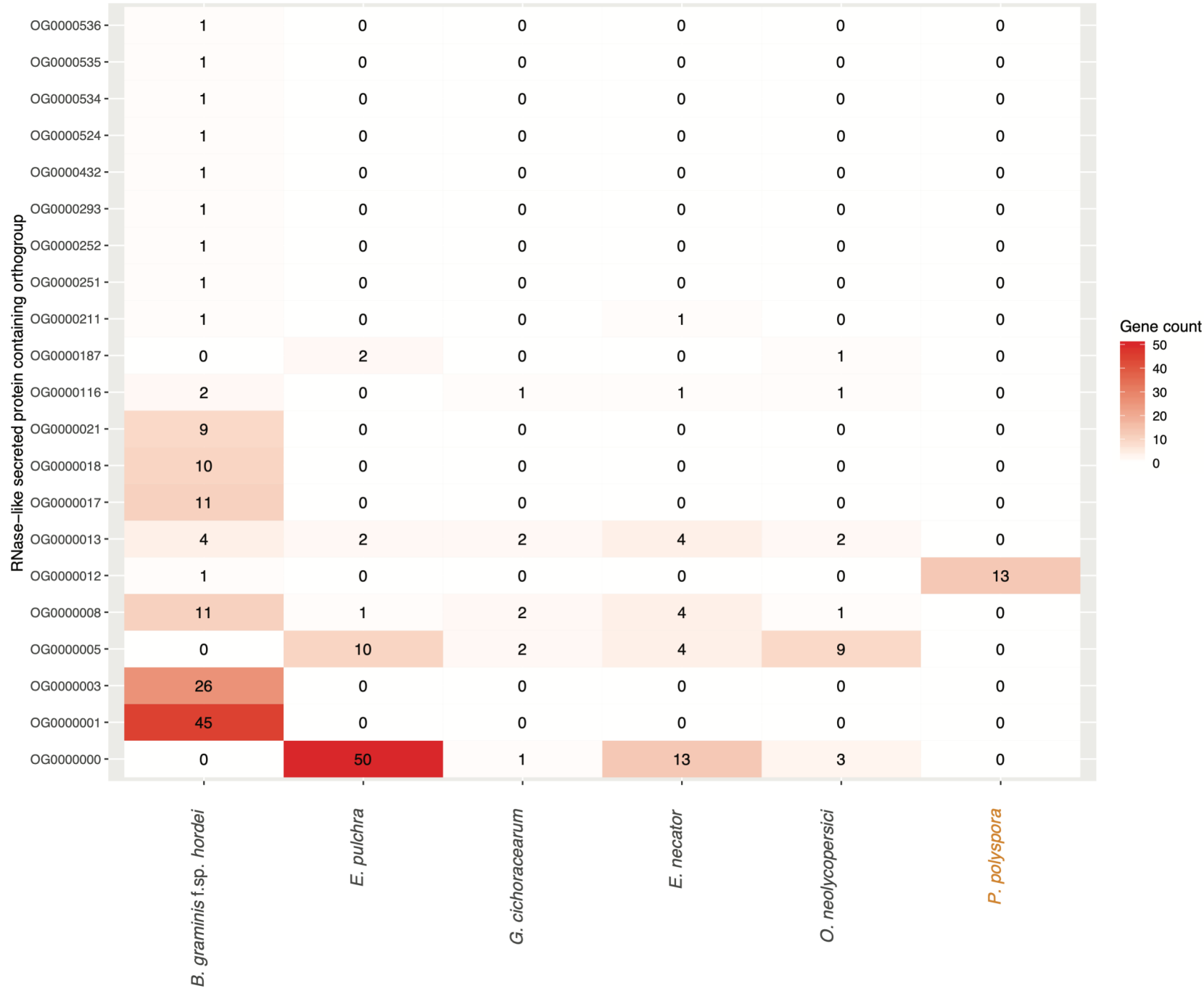

Supplement: FIG S7 [file mBio.01692-19-sf007.pdf]
