# Supplementary material for: The Parauncinula polyspora Draft Genome Provides Insights into Patterns of Gene Erosion and Genome Expansion in Powdery Mildew Fungi
Source: mBio. 2019 Sep 24;10(5):e01692-19. doi: 10.1128/mBio.01692-19 (PMC6759760; doi:10.1128/mBio.01692-19)
Supplement: FIG S3 [file mBio.01692-19-sf003.pdf]

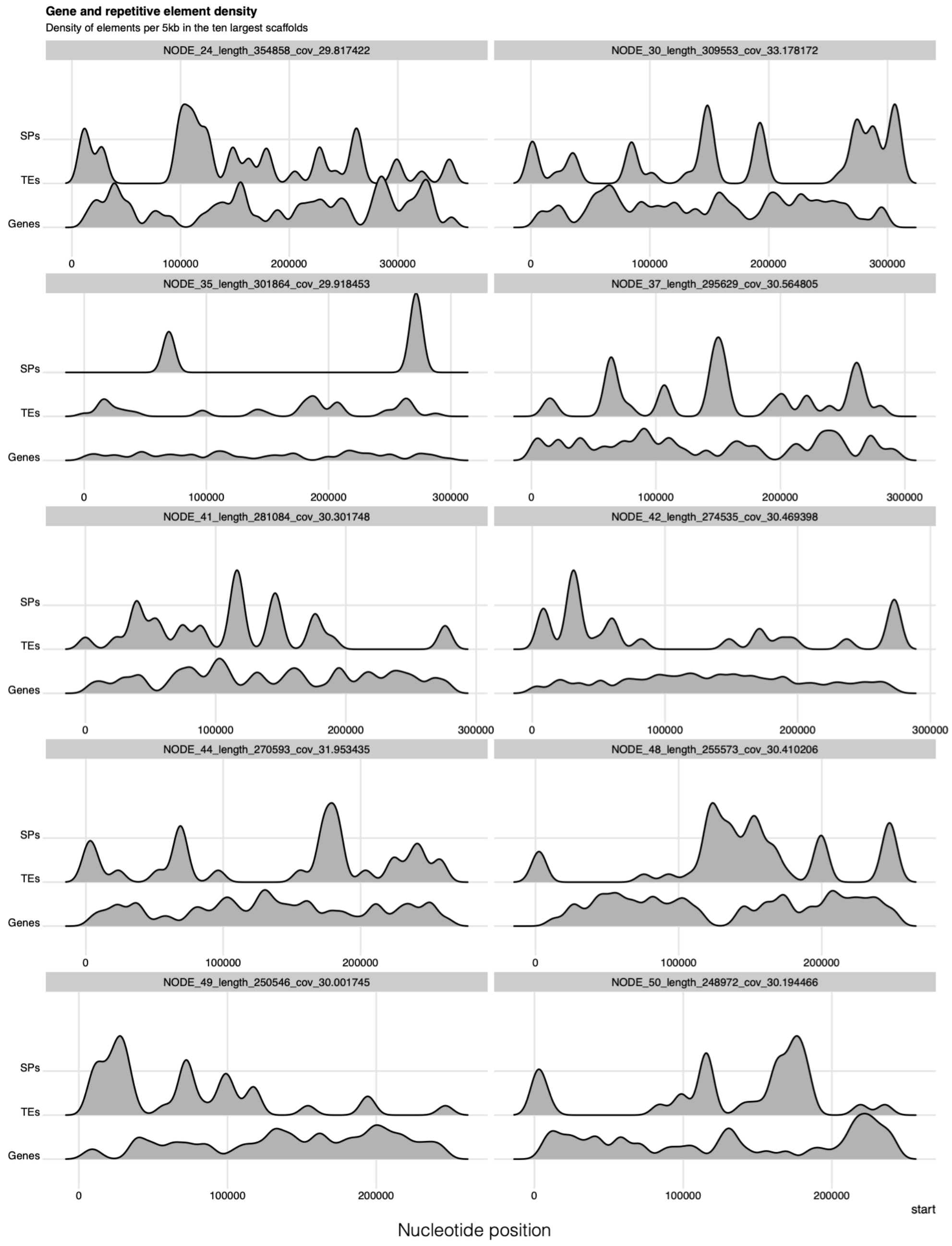
Gene and repetitive element density
Density of elements per 5kb in the ten largest scaffolds
NODE_24_length_354858_cov_29.817422
NODE_30_length_309553_cov_33.178172
NODE_35_length_301864_cov_29.918453
NODE_37_length_295629_cov_30.564805
NODE_41_length_281084_cov_30.301748
NODE_42_length_274535_cov_30.469398
NODE_44_length_270593_cov_31.953435
NODE_48_length_255573_cov_30.410206
NODE_49_length_250546_cov_30.001745
NODE_50_length_248972_cov_30.194466
SPs
TEs
Genes
0
100000
200000
300000
start
Nucleotide position
